# Automated Intracellular Immunofluorescence Staining Enabled by Magnetic 3D Mixing in a Modular Microfluidic Platform

**DOI:** 10.3390/bios16020120

**Published:** 2026-02-13

**Authors:** Zhengyi Zhang, Mengyu Wang, Runtao Zhong, Yingbo Zhao, Yeqing Sun

**Affiliations:** Institute of Environmental Systems Biology, College of Environmental Science and Engineering, Dalian Maritime University, Dalian 116026, China; zhengyi-zhang@dlmu.edu.cn (Z.Z.); wangmengyu@dlmu.edu.cn (M.W.); rtzhong@dlmu.edu.cn (R.Z.); yingbo.zhao@illumina.com (Y.Z.)

**Keywords:** magnetic field simulation, immunomagnetic beads (IMBs), microfluidic, immunofluorescence (IF) staining

## Abstract

Traditional sample preparation for flow cytometry is often labor-intensive, operator-dependent, and reagent-consuming, limiting its suitability for automated and point-of-care biosensing applications. To address these challenges, this study presents a functional modular microfluidic system integrating immunomagnetic beads (IMBs) to enable automated intracellular immunofluorescence (IF) staining. The modular microfluidic platform is enabled by a dynamically actuated three-dimensional magnetic field that couples with IMBs within a microfluidic reaction chamber, requiring only one-dimensional magnet translation to induce effective three-dimensional bead motion. This magnetic–chip cooperative strategy significantly enhances microscale mixing and cell capture, facilitating automated immunostaining of the radiation biomarker in CD4^+^ cells. Finite element simulations were employed to guide magnetic field design by analyzing magnetic force distributions and identifying key parameters, including magnet material, size, spatial arrangement, and chip–magnet distance. Experimental validation using CD4^+^ cell capture confirmed the effectiveness of the magnetic mixing strategy, revealing ***∇B·B*** as the critical design parameter. An N52 NdFeB magnet (6 mm diameter, 10 mm height) positioned within 2.2 mm of the chamber centerline stably retained IMBs at flow rates below 200 µL/min. Under optimized conditions (magnet translation speed of 8 mm/s and a 15 min mixing duration), a maximum cell capture efficiency of 86% was achieved. Subsequent automated γH2AX IF staining demonstrated a strong linear dose–response relationship (R^2^ > 0.9) in mean fluorescence intensity. This study demonstrates a robust and scalable strategy for automating complex IF staining workflows, highlighting the potential of magnetic-field-assisted microfluidic platforms for biosensing applications requiring reliable intracellular biomarker detection.

## 1. Introduction

Intracellular immunofluorescence (IF) staining combined with flow cytometry has become a fundamental technique for single-cell functional analysis in immunology, oncology, and radiobiology [1]. By enabling quantitative detection of intracellular and nuclear proteins at the single-cell level, this approach has emerged as an indispensable tool in basic research, translational studies, and clinical investigations [2]. Consequently, flow cytometry has been extensively applied to the interrogation of signaling pathways, cellular stress responses, and the analysis of complex biomarker profiles [3]. Conventional intracellular IF protocols rely on a series of labor-intensive manual procedures, including fixation, permeabilization, repeated centrifugation, and multiple washing steps to remove unbound antibodies [2,4]. These processes not only prolong assay time and increase operator dependence but also require sophisticated laboratory infrastructure, rendering them incompatible with rapid, automated, or resource-limited diagnostic settings [5]. These limitations greatly restrict the broader deployment of intracellular IF in scenarios that demand robust and scalable automation, such as POCT-oriented analytical workflows and aerospace-related applications.

To meet the increasing demand for rapid, miniaturized, and automated bioanalytical assays, microfluidics (lab-on-a-chip, LOC) has been widely recognized as a promising technological approach. By integrating complex laboratory workflows into compact and highly controllable microscale devices, LOC technologies offer distinct advantages, including low reagent consumption, high throughput, excellent biocompatibility, and precise operational control [6,7]. Owing to these features, LOC technologies exhibit substantial potential for rapid sample pretreatment and analytical applications and have been widely explored in diverse fields, including disease diagnostics, chemical screening, cell culture, and cell separation [8,9,10]. Within this context, efficient and reliable strategies for target manipulation and enrichment are essential for maximizing the performance of LOC-based assays. Immunomagnetic bead (IMB)-based methods have attracted considerable attention as a simple yet highly effective approach for purification and sorting applications [11]. In IMB-based assays, magnetic beads functionalized with antibodies or other biomolecules enable selective binding to target entities. The resulting bead–target complexes can be magnetically manipulated for washing and separation and subsequently re-dispersed for downstream analytical or biological processes [12]. The integration of microfluidic chips with IMBs has demonstrated distinct advantages in applications such as cell staining, cell sorting, and solution mixing. For example, Fatma Betul et al. [13] achieved manual staining of multiple cell types with extremely low reagent consumption, minimal waste generation, low cost, and operational simplicity, while enabling cell recovery for downstream applications. In another study, Lyu Chuan et al. [14] developed an automated and miniaturized magnetic digital microfluidic–chemiluminescence immunoassay (MDMF-CLIA) system that enabled the sensitive and specific detection of two cardiac biomarkers, cardiac troponin I (cTnI) and myoglobin (Myo), within 10 min, exhibiting favorable linearity, repeatability, and accuracy. Together, these advances underscore the potential of magnetically assisted microfluidic systems to enable efficient and automated bioanalytical workflows.

However, within microfluidic channels—typically on the millimeter or even micrometer scale—achieving rapid, efficient, and homogeneous mixing between cells and reagents (e.g., magnetic beads and antibodies) remains a long-standing technical challenge [15,16]. Inefficient mixing often leads to non-uniform staining, weak fluorescence signals, and poor reproducibility during downstream detection [2]. At present, strategies to enhance on-chip fluid mixing can be broadly categorized into active and passive approaches [17]. Among them, active mixing schemes that utilize magnetic forces to manipulate IMBs have been widely adopted in microfluidic technologies due to their effectiveness and controllability [18]. Numerous studies combining numerical simulations with experimental validation have demonstrated the advantages and broad applicability of magnetic beads in improving mixing efficiency on microfluidic chips. Nevertheless, relatively limited attention has been paid to the impact of enhanced mixing on intracellular protein staining, and many reported systems rely on complex magnetic actuators [19,20,21]. In addition, although many magnetically integrated microfluidic platforms have successfully enabled on-chip immunoassays, the majority of these efforts have focused on CLIA detection or cell surface marker analysis [13,14,22]. By contrast, the automation of intracellular IF staining—particularly for nuclear proteins that require sequential fixation and permeabilization—remains a relatively underexplored frontier in LOC-based biosensing systems.

To address these challenges, a cooperative three-dimensional (3D) magnetic mixing strategy integrated with a specifically designed microfluidic platform was developed to enable automated intracellular IF staining of nuclear proteins. The resulting stained cell suspension remains in a single-cell state and is directly compatible with flow cytometric analysis. The core design concept is to achieve efficient 3D mixing of magnetic beads within the reaction chamber using a simplified one-dimensional linear magnetic actuation scheme. By strategically configuring the spatial arrangement of magnets, IMB–cell complexes are driven to execute cyclic 3D trajectories spanning the entire depth of the chamber, thereby enhancing mass transfer and ensuring homogeneous reagent–cell interactions. It should be clarified that the present work focuses on a functional microfluidic system composed of multiple coordinated modules, including fluid handling and magnetic actuation, rather than a fully integrated end-to-end laboratory-on-a-chip device.

To evaluate the feasibility and robustness of this automated intracellular staining microfluidic platform, phosphorylated histone H2AX (γH2AX) was selected as a representative nuclear protein marker. γH2AX, a phosphorylated form of the H2AX protein in DNA histone cores, is specifically induced at sites adjacent to DNA double-strand breaks (DSBs) and is widely recognized as an early indicator of DNA damage [23,24]. Because ionizing radiation (IR) and many chemotherapeutic agents used in cancer treatment generate DSBs, γH2AX has been extensively employed as a key determinant of cellular radiation responses in both normal and cancer cells [25,26]. In this context, CD4^+^ T lymphocytes (CD4^+^ cells) were chosen as the model cell type because they constitute a well-characterized and clinically relevant immune cell population that exhibits sensitive and reproducible DNA damage responses [27,28]. Moreover, γH2AX staining in CD4^+^ cells has been widely adopted as a representative nuclear protein readout in immunology, oncology, and radiobiology studies [29]. Although conventional IF microscopy enables visualization of γH2AX foci at the single-cell level, it is inherently limited by low throughput and subjective scoring [30,31]. Compared to immunofluorescence microscopy, flow cytometry is relatively more accessible and capable of multiparametric data acquisition and expediting the process of comparing specific cellular subsets in an efficient manner [4,32]. These features make flow cytometry particularly well suited for quantitative single-cell analysis and compatible with automated LOC-based workflows.

In our previous work [33], we proposed an on-chip magnetic three-dimensional mixing mechanism based on eccentrically rotating upper and bottom circular plates embedded with magnets arranged in a staggered formation (ec2MagRotors). Motor-driven rotation of the plates generated a periodically varying 3D magnetic field, enabling effective mixing between magnetic beads and cells and thereby improving immunomagnetic capture efficiency. However, this system relied on manual reagent loading and a multi-chamber workflow assisted by an additional one-dimensional translation module, in which bead–cell complexes were sequentially transported across multiple reaction zones. Such a design increased operational complexity and limited automation, robustness, and parallel sample processing. Motivated by the need to simplify the staining workflow and enhance system-level automation and scalability, the present study fundamentally revises both the magnetic actuation strategy and the microfluidic architecture. Specifically, numerical simulations were employed to elucidate the interplay between magnetic field gradients and hydrodynamic drag forces under a reconfigured magnetic field geometry. Guided by the simulation results, a dynamically actuated three-dimensional magnetic field was established to simultaneously enable efficient on-chip mixing and stable bead–cell retention within a single reaction chamber, eliminating the need for translation-assisted multi-chamber operation. The capture efficiency of CD4^+^ cells was used as an indirect indicator of mixing performance. Based on this magnetic field configuration, an integrated microfluidic approach for automated intracellular immunofluorescence staining of γH2AX in CD4^+^ cells was developed. This work demonstrates that a magnetically assisted 3D mixing microfluidic platform can substantially enhance mixing efficiency while reproducing the analytical accuracy of conventional manual IF protocols. The presented modular microfluidic platform provides a generalizable framework for automated intracellular and nuclear protein staining coupled with flow cytometric analysis, thereby serving as a key enabling module for the future translation of rapid IF-based assays into biosensing and POCT-oriented analytical workflows.

## 2. Materials and Methods

### 2.1. Design and Fabrication of the Microfluidic Chip

**Figure 1:**
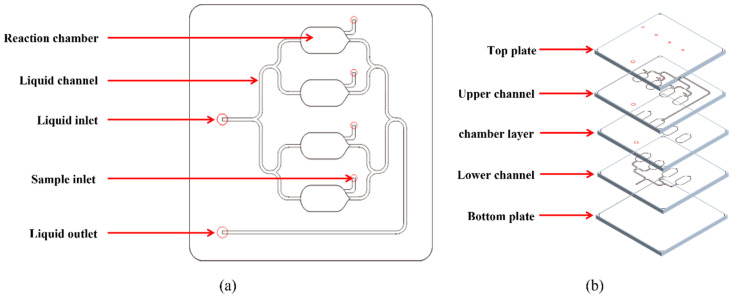
Schematic diagram of microfluidic chip. (**a**) Overall display of the chip. (**b**) Layered chip display. (The red circles represent the perforations, indicating the inlet and outlet positions for the solution).

The microfluidic chip designed in this study comprises an inlet, a separation channel, four parallel liquid chambers, and an outlet, each equipped with a sampling port (Figure 1). The overall chip dimensions form a 35 × 35 mm rectangle, featuring a channel width of 0.5 mm. Each reaction chamber measures 4 mm in width and 10 mm in length, with an inter-chamber spacing of 4 mm. The chip with a single discharge chamber is composed of five layers: a cover plate (1 mm thick), an outlet channel layer (0.2 mm), a chamber layer (2 mm), an injection channel layer (0.2 mm), and a base plate (1 mm). The volume of each chamber is approximately 80 μL. In this multilayer design, the lower channel layer functions as the solution inlet, while the upper channel layer serves as the outlet. The intermediate chamber layer defines the reaction chamber where immunomagnetic beads and target cells are retained, mixed, and subjected to intracellular IF staining. Separating the inlet and outlet into different layers facilitates more complete solution exchange within the chamber, while the chamber height provides sufficient space for three-dimensional bead–cell motion and magnetic mixing.

To assemble the five layers, poly (methyl methacrylate) (PMMA) and double-sided adhesive film (Adhesives Research, Inc., Glen Rock, PA, USA) were cut using a CO_2_-laser cutter (YoungChip, Inc., Hangzhou, China). Two layers of double-sided adhesive films between the PMMA layers were cut with the same design as the middle channel plate. After alignment and assembly, the chip was firmly bonded by using an air press to obtain a uniform seal.

### 2.2. Design of the Magnetic Field Structure for 3D Motion of Magnetic Beads

**Figure 2:**
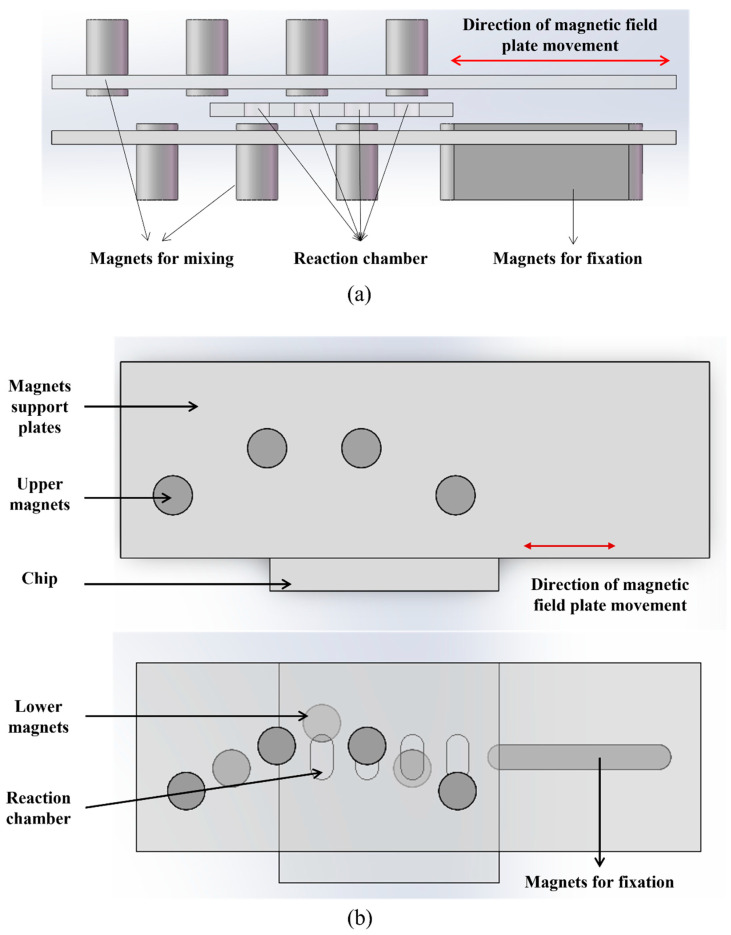
Schematic diagram of Magnetic Field Device. (**a**) Front view. (**b**) Vertical view. (The red two-way arrow indicates the direction of motion of the magnetic field plate).

The 3D mixing magnetic field system comprises two main components: a mixing magnet area and a fixed magnet area. The mixing magnet area consists of two layers, each containing magnets arranged in a V-shaped configuration, with the upper and lower layers oriented in a staggered arrangement. The fixed magnet area is composed of a rectangular magnet positioned in the lower layer. The microfluidic chip is positioned between two magnetic plates, as illustrated in Figure 2.

**Figure 3:**
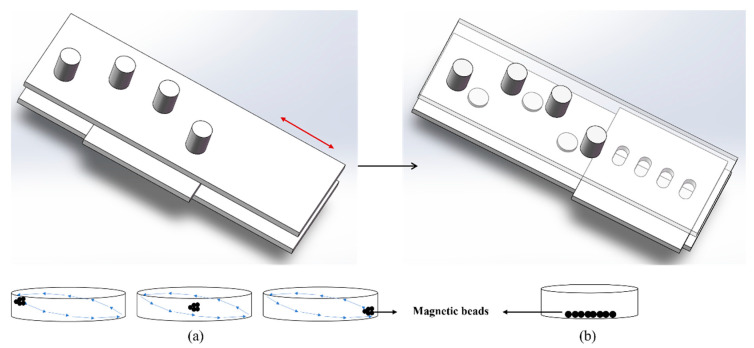
Schematic diagram of two states of 3D magnetic field. (**a**) The reaction (3D mixing) phase diagram. (**b**) The liquid exchange (MB fixation) phase diagram. (The red two-way arrow indicates the direction of motion of the magnetic field plate and the black right arrow in the middle of the figure represents the transition between the two phases).

Taking one reaction chamber as an example, the V-shaped arrangement of the external magnets in the mixing zone—combined with the staggered layout of the two magnet layers—enables magnetic beads to be attracted in multiple directions within the chamber even when the magnets are driven with one-dimensional motion. As a result, the beads undergo 3D movement inside the chamber. Through the reciprocating motion of the magnetic field, the magnetic beads perform periodic 3D trajectories, thereby achieving efficient mixing and incubation (Figure 3a).

When the reaction chamber of the microfluidic chip is positioned above a rectangular magnet within a fixed magnetic zone, the magnetic beads in the liquid chamber can be magnetically captured and immobilized against one side of the chamber. This configuration enables the beads to withstand the hydrodynamic drag forces generated during fluid exchange, ensuring that they remain firmly attached and are not flushed out of the reaction chamber by liquid flow. The retained beads are then available for subsequent steps, such as antibody incubation after fluid replacement (Figure 3b).

### 2.3. Force Analysis of Magnetic Beads and Configuration of Numerical Simulation Model

When magnetic beads are introduced into the reaction chamber of the microfluidic chip and external magnets are positioned outside the chamber, the beads are subjected to multiple forces within the microfluidic environment. Among these forces, the dominant contributions arise from the magnetic force (**F_m_**) generated by the external magnetic field and the hydrodynamic drag force (**F_d_**) induced by fluid flow. Other forces, such as gravitational and buoyant forces acting on the beads, are typically negligible due to the small size and low mass of the magnetic beads [34].

The acting forces vary under different operational conditions:

Moving magnet, static fluid: When the external magnet moves and the fluid is stationary, **F_d_** approaches zero. The bead primarily follows the magnet due to **F_m_**.

Static magnet, flowing fluid: When the magnet is stationary and the fluid flows, a stationary bead experiences both a constant **F_m_** (holding it in place) and **F_d_** from the fluid flow.

In microfluidic devices, the characteristic dimensions of flow channels and reaction chambers are typically on the order of micrometers to millimeters, resulting in Reynolds numbers (**Re**) well below 2000 and thus laminar flow conditions. Under such conditions, according to Stokes’ law, the viscous drag force **F_d_** exerted on a magnetic bead suspended in a flowing fluid can be expressed as follows:

According to Stokes’ law, the viscous drag force exerted on the magnetic beads during fluid motion is

(1) $$F_{d}=6\pi \eta Rv$$

in which ***R*** is the radius of the magnetic bead, and ***ν*** represents the relative velocity between the magnetic bead and the fluid. When the external magnetic field is stationary, the velocity of the magnetic bead is zero, and ***ν*** corresponds to the flow velocity of the liquid. ***η*** denotes the dynamic viscosity of the liquid. It is important to note that Stokes’ drag law is applicable only under laminar flow conditions [35].

According to classical electromagnetic theory, the magnetic force exerted on a magnetic bead is given by

(2) $$F_{m}=V\cdot X_{m}\cdot \left(H\cdot \nabla \right)B$$

Owing to the superparamagnetic property of the beads, ***χ_m_*** << 1. The magnetic flux density ***B*** is related to the magnetic field ***H*** by [36]

(3) $$B=\mu_{0}\left(H+M\right)=\left(\chi_{m}+1\right)\mu_{0}\cdot H$$

Thus, the magnetic force can be reformulated as

(4) $$F_{m}=\frac{V\cdot X_{m}}{\mu_{0}}\cdot \left(B\cdot \nabla \right)B$$

Here, ***B*** denotes the magnetic flux density, ***H*** the magnetic field strength, ***M*** the magnetization, ***m*** the magnetic moment of a single magnetic bead, ***χ_m_*** the magnetic susceptibility of the magnetic bead, ***V*** the volume of the magnetic bead, and ***μ*_0_** the vacuum’s magnetic permeability. For a given type of MBs, ***V***, ***χ_m_***, and ***μ*_0_** are fixed constants. Therefore, the term ***∇B·B*** becomes the key determinant of the magnetic force [34]. In this expression, ***∇B*** represents the gradient of the magnetic field (i.e., the spatial rate of change in the magnetic field), and ***B*** is the magnetic flux density. The unit of ***∇B·B*** is kg^2^·m^−1^·s^−4^·A^−2^.

During the bead immobilization stage, magnetic beads are subjected to both fluid force from buffer exchange flow and magnetic force. To ensure that beads remain in the reaction chamber after cell capture, it is necessary to calculate the fluid force at different flow rates and the corresponding ***∇B·B*** values, thereby selecting an appropriate buffer exchange velocity. Four different flow rates were selected for simulation, as detailed in Table 1.

Based on preliminary experiments and empirical observations during system development [33], three parameters were found to exert the most significant influence on magnetic bead motion and retention, and were therefore prioritized for numerical investigation in this study. Specifically, the parameters include (1) the material and dimensions of the magnets, (2) the distance between the upper/lower magnets and the chip reaction chamber, and (3) the arrangement range and spacing of the magnets. Accordingly, a series of parameters—including magnet material, height, and distance from the reaction chamber—were designed for evaluation, as listed in Table 1.

The purpose of the three-dimensional (3D) mixing module is to achieve efficient 3D mixing between magnetic beads and the sample. The constructed 3D magnetic field should provide sufficient magnetic force and a well-distributed magnetic force profile to ensure effective mixing [18,37]. Finite element multiphysics analysis of the designed magnetic field was performed using the Magnetic Fields module in COMSOL Multiphysics 6.3. To simplify the model and reduce computational cost, a two-dimensional plane was used for static field analysis, while a three-dimensional model was applied to analyze the 3D magnet arrangement. The selected physical interfaces included Magnetic Fields, No Currents (MFNC) to simulate the magnetic field and force distribution, and Laminar Flow (SPF) to simulate the fluid flow inside the chip. The study was conducted under steady-state conditions, with the mesh set to Extra Fine. Detailed parameters for the physical interfaces are provided in Table 2.

### 2.4. 3D Magnetic Mixing-Assisted Capture of CD4^+^ Cells Using Immunomagnetic Beads

Cultured CD4^+^ cells were mixed and captured with Dynabeads^®^ FlowComp™ Human CD4 (11361D, Thermo Fisher, Inc., Waltham, MA, USA) in the designed 3D magnetic field microfluidic system according to the manufacturer’s protocol. The cell-bead binding efficiency (Cell capture efficiency) was used to evaluate the rationality of the magnet arrangement design. Experiments were conducted to investigate the effects of different magnetic field translation speeds and mixing durations. A control group was set up using the standard tube-based mixing method on a shaker (as described in the protocol), while the experimental group employed the 3D mixing magnetic field within the microfluidic system.

The bead preparation protocol was as follows:

1. Resuspend the beads in the vial (i.e., vortex for >30 s, or tilt and rotate for 5 min).

2. Transfer the desired volume of beads to a tube.

3. Add the same volume of PBS-BSA Buffer (SL6730, Coolaber, Inc., Beijing, China) from step 2, or at least 1 mL, and resuspend.

4. Place the tube in a magnet for 1 min and discard the supernatant.

5. Remove the tube from the magnet and resuspend the washed beads in the same volume of PBS-BSA Buffer as the initial volume of beads (step 2).

Cell-Bead Mixing and Capture:

1. Transfer 100 μL counted CD4^+^ single-cell suspension to a tube and add 5 μL FlowComp™ Human CD4 Antibody (11361D, Thermo Fisher).

2. Mix well and incubate for 10 min at 2 °C to 8 °C.

3. Wash by adding 2 mL PBS-BSA Buffer and centrifuge for 8 min at 350× *g*.

4. Remove the supernatant and resuspend in 1 mL PBS-BSA Buffer.

5. Add the prepared FlowComp™ Dynabeads^®^ (11361D, Thermo Fisher) and mix well.

6. For the control group, the mixture was incubated for 20 min at room temperature with end-over-end mixing or on a shaker. For the experimental group, the cell-bead mixture was loaded into the reaction chamber of the microfluidic chip, and the 3D mixing magnetic field was activated, driving the beads with a defined linear velocity in a one-dimensional reciprocating motion for 20 min at room temperature.

7. After mixing, the supernatant was collected for cell counting. In the control group, PBS-BSA was added to a final volume of 500 μL, mixed by pipetting, placed on a magnet for 2 min, and the supernatant was taken to count unbound cells. In the experimental group, the stationary magnet was held beneath the chip’s reaction chamber for 2 min to immobilize the cell-bead complexes, and the solution in the chamber was collected to count unbound cells.

### 2.5. Automated Intracellular IF Staining of γH2AX

**Figure 4:**
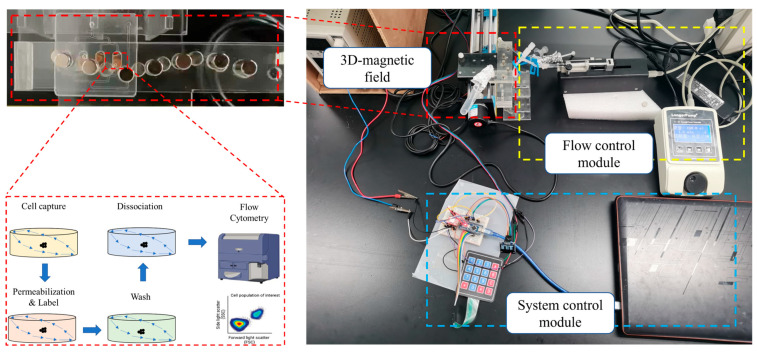
Schematic diagram of the microfluidic system and the experimental procedure.

Cell culture and UVC irradiation were performed following previously described methods [36], with irradiation doses in this study set at 0, 24, 48, 96, and 192 J/m^2^. After mixing MBs with cells for 15 min according to the protocol in Section 2.4, the fixed magnet was positioned beneath the chip reaction chamber to immobilize the magnetic beads at the bottom. During the bead immobilization phase, the solution in the reaction chamber was exchanged via fluidic channels. The first solution introduced consisted of a permeabilization buffer (00-5523-00, Thermo Fisher) mixed with Anti-γ-H2AX (phospho S139) antibody (ab195188, Abcam, Inc., Cambridge, MA, USA) at a dilution of 1:5000. Following infusion, the mixture was incubated for 30 min under a stationary magnetic field. Subsequently, the chamber was flushed with permeabilization buffer alone and mixed for 5 min; this washing step was repeated twice. Finally, magnetic bead FlowComp™ Release Buffer (11361D, Thermo Fisher) was introduced and mixed for 10 min to release the beads. The labeled CD4^+^ cells can be analyzed immediately by flow cytometry to determine fluorescence intensity, or stored at 4 °C protected from light. A schematic of the microfluidic system and the experimental procedure is shown in Figure 4.

### 2.6. Data Analysis

Under an inverted microscope, CD4^+^ cells were counted using a cell counting plate. To evaluate the capture efficiency of magnetic beads on CD4^+^ cells, calculations were made based on the formula

(5) $$E\%=\frac{N_{t}-N_{u}}{N_{t}}\times 100\%\;$$

in which ***E*%** represents the capture efficiency of cells, ***N_t_*** total number of cells, and ***N_u_*** the number of uncaptured cells. The experimental results were presented as mean ± standard deviation (±SD). The intergroup differences were assessed using one-way analysis of variance (ANOVA) followed by the Tukey post hoc test. A *p*-value of 0.01 < *p* ≤ 0.05 was considered statistically significant (*), 0.001 < *p* ≤0.01 was considered highly significant (**), and *p* ≤ 0.001 was regarded as extremely significant (***). Statistical analysis was performed using Origin2024 software (OriginLab Corporation, Northampton, MA, USA).

The fluorescence intensity of the stained CD4^+^ cells was measured using a flow cytometer (BD FACSCalibur™, BD Life Sciences, Inc., San Jose, CA, USA ). All the flow cytometry data were analyzed using FlowJo v10.8 Software (BD Life Sciences). A total of 10,000 CD4^+^ cells were collected from the FL1 channel to obtain the mean fluorescence intensity (MFI). The expression level of γH2AX in CD4^+^ cells was represented by the relative fluorescence intensity (RFI), calculated as the MFI of the experimental group divided by that of the negative control group. Subsequently, a linear regression was performed with radiation dose as the x-axis and the RFI of γH2AX as the y-axis to establish a dose–response curve.

## 3. Results and Discussion

### 3.1. Relationship Between F_d_ and **∇B·B** Under Different Flow Rates

**Figure 5:**
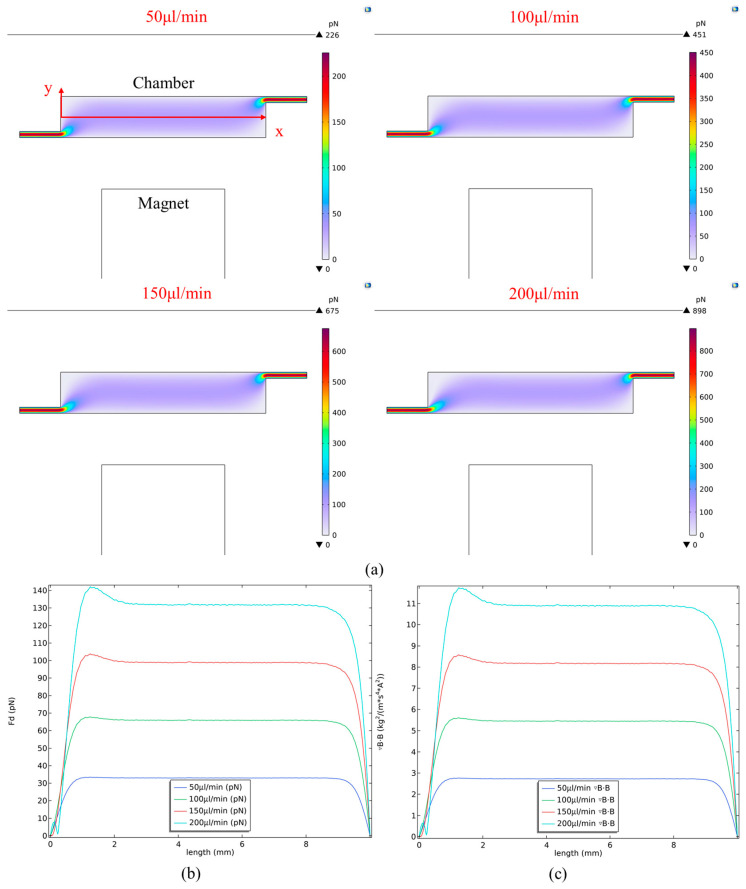
**F_d_** and corresponding ***∇B·B*** at different flow rates. (**a**) Distribution of **F_d_** within the reaction chamber. (**b**) Magnitude of **F_d_** along the chamber centerline. (**c**) Magnitude of corresponding ***∇B·B*** along the chamber centerline.

Based on the dimensions of the chip reaction chamber and the set flow rates, the **Re** values at different flow velocities were calculated, as shown in Table 3. The results demonstrate that the Re values along the centerline inside the chip reaction chamber are all well below 2000, which is indicative of typical laminar flow. Therefore, the magnitude of the viscous drag force corresponding to each flow rate could be calculated using Equation (1), with all calculations performed along the centerline (Figure 5a) of the reaction chamber. The fluid moves at a higher speed in the narrow channels, resulting in a greater viscous drag force on the magnetic beads. The maximum viscous drag force at a flow rate of 200 μL/min reached 898 pN. Upon entering the reaction chamber, the cross-sectional area increases, causing a sharp decrease in linear velocity and a corresponding drop in fluid drag. Along the centerline in the *x*-direction inside the chip reaction chamber, the maximum **F_d_** remained around 142 pN (Figure 5b). The **F_d_** were then substituted into Equation (4) to derive the corresponding magnetic field gradient term ***∇B·B*** acting on the MBs (Figure 5c). The magnetic susceptibility (***χ_m_***) of the beads, taken from the measurements reported by G. Fonnum et al. [38], is 0.756 (dimensionless). The calculated ***∇B·B*** values for the different flow rates are summarized in Table 3.

Based on the results, the magnetic field gradient term ***∇B·B*** generated by the magnet must theoretically exceed 2.76 along the centerline of the reaction chamber to overcome the **F_d_** on the MBs at a flow rate of 50 μL/min and thus retain the beads within the chamber. Higher flow rates require a greater ***∇B·B*** to counteract the increased fluid drag. During solution exchange, the beads are held by the magnet against one side of the chamber to keep cells in place for sequential reagent reactions. The maximum flow velocity and consequently the highest **F_d_** occur along the centerline of the chamber. In the mixing phase, the preliminary magnet arrangement is placed above and below the chamber. The one-dimensional movement of the magnetic field causes the vertical (*y*-direction) field from the upper and lower magnets to shift alternately. The centerline represents the furthest position in the *y*-direction that must be considered for a single-sided magnet, beyond which beads would be influenced by the opposing magnet’s field. Therefore, all subsequent analysis in this study is performed at the centerline of the reaction chamber.

### 3.2. Effect of Magnet Size and Material on Magnetic Field Strength and Force Distribution

**Figure 6:**
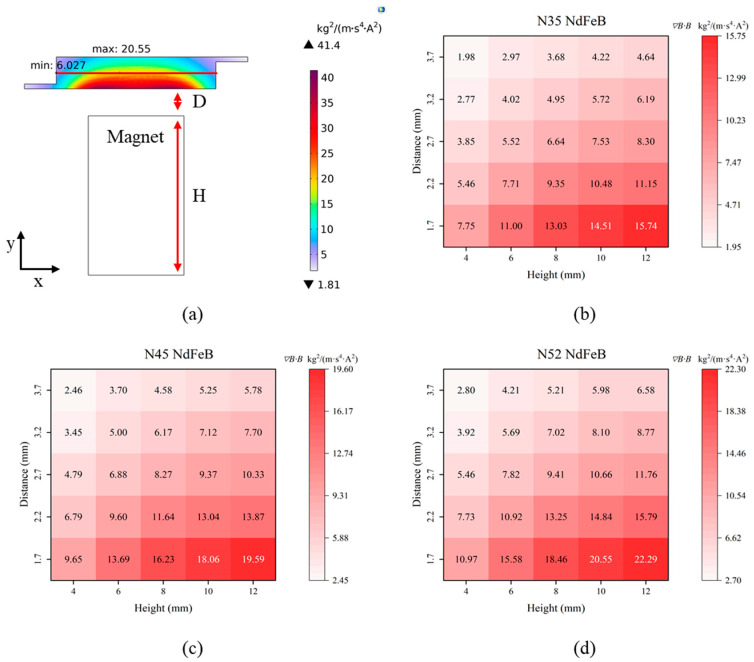
Simulated ***∇B·B*** values along the reaction chamber centerline for different magnet materials, distances (D), and heights (H). (**a**) Minimum and maximum ***∇B·B*** along the centerline for an N52 magnet with D = 0.5 mm and H = 10 mm. (**b**) Distribution of maximum ***∇B·B*** along the centerline for an N35 magnet. (**c**) Distribution of maximum ***∇B·B*** along the centerline for an N45 magnet. (**d**) Distribution of maximum ***∇B·B*** along the centerline for an N52 magnet.

In the study of magnet dimensions and materials, neodymium–iron–boron (NdFeB) permanent magnets—currently recognized as the strongest type—were selected, with performance grades of N35, N45, and N52. Based on the size of the microfluidic chip reaction chamber, cylindrical magnets with a diameter of 6 mm were used for simulation analysis. The magnet height was set to H, with values of 4, 6, 8, 10, and 12 mm. The closest distance between the magnet and the bottom of the chip reaction chamber was set to 1.7 mm, determined by the combined thickness of the chip channel and the substrate (1.2 mm) plus a clearance of at least 0.5 mm. Additional distances were increased in increments of 0.5 mm, with the farthest distance being 3.7 mm. The analysis of the magnetic field gradient term ***∇B·B*** along the *y*-direction followed the same approach described in Section 3.1, and this applies to all subsequent experiments. A representative result from a single analysis is shown in Figure 6a.

Figure 6b, Figure 6c and Figure 6d present the results for N35, N45, and N52 NdFeB magnets under varying parameters of distance (D) and height (H), respectively. The corresponding simulation plots for each case are provided in Figures S1–S3. The results indicate that the magnetic field strength increases with greater magnet height, while it gradually attenuates within the reaction chamber as the magnet moves farther away. Based on the analysis of the ***∇B·B*** values required for different flow rates (Table 2), to overcome the **F_d_** at a flow rate of 200 μL/min, the distance between the magnet and the chip should not exceed 1 mm (corresponding to the 2.2 mm datum in the figure), and the magnet height must be at least 8 mm. Notably, for N35 NdFeB magnets, the theoretical condition for overcoming the **F_d_** is only satisfied at a distance of 0.5 mm and a height of 8 mm or greater.

**Figure 7:**
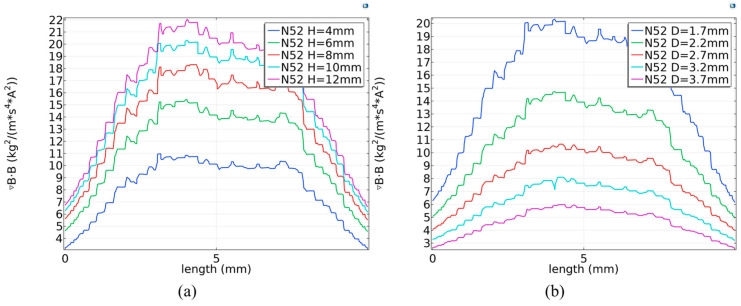
Comparison of ***∇B·B*** variations along the reaction chamber centerline for different parameters. (**a**) Comparison of ***∇B·B*** for an N52 NdFeB magnet at D = 1.7 mm across varying H. (**b**) Comparison of ***∇B·B*** for an N52 NdFeB magnet at H = 10 mm across varying D.

Theoretically, larger magnet dimensions yield stronger magnetic forces and a broader range. Furthermore, research indicates that the geometric aspect ratio (height-to-diameter) of a cylindrical magnet considerably influences its magnetic performance [39]. To enhance the magnetic field while keeping the diameter constant at 6 mm and the distance at 1.7 mm, the height of the N52 magnet was increased from 4 mm to 12 mm. As demonstrated by the simulation results (Figure 6d and Figure 7a), the maximum value of ***∇B·B*** along the centerline of the reaction chamber increased from 10.97 to 22.29—a 103% improvement—when the magnet height was raised from 4 mm to 12 mm. Despite this overall growth, the rate of improvement diminished with greater heights: for example, a 42% increase was observed when going from 4 mm to 6 mm, compared to only 8.5% from 10 mm to 12 mm. Based on the combined magnetic and hydrodynamic simulation results, a height of 10 mm was chosen. Consequently, the magnetic field was constructed using a cylindrical N52 NdFeB magnet with dimensions of 6 mm in diameter and 10 mm in height.

### 3.3. Influence of Magnet–Chip Distance on Magnetic Field Gradient and Bead Actuation

The microfluidic chip has a thickness of 4.4 mm. To ensure operational safety, the minimum distance between the upper and lower magnets was set to 5.4 mm, corresponding to a 0.5 mm gap from each magnet to the outer chip surface (or 1.7 mm from the magnet to the inner edge of the reaction chamber). This prevents friction or collision due to assembly tolerances. By comparing the magnetic performance across five gap distances ranging from 0.5 mm to 2.5 mm (Figure 6 and Figure 7b), ***∇B·B*** was found to increase significantly as the gap decreased. Simulation results show that, for a 10 mm high N52 NdFeB magnet, the peak ***∇B·B*** value along the chamber centerline reached 20.55 at a 1.7 mm gap, representing increases of 93% and 244% compared to the values at 2.7 mm (10.66) and 3.7 mm (5.98), respectively. It is evident that the magnetic force weakens as the magnet moves away from the chip. At a distance of 3.7 mm from the reaction chamber, the ***∇B·B*** product along the centerline is very close to 5.76—the value required to counteract the **F_d_** at a flow rate of 100 µL/min. Therefore, if the magnet is 2.5 mm or farther from the outer chip surface, MBs risk being flushed out during solution exchange. Although a smaller gap yields a stronger magnetic field, practical fabrication and assembly tolerances must be considered. A distance of less than 1 mm from a single magnet to the chip surface is deemed appropriate. Consequently, a final spacing of 5.4 mm between the upper and lower magnet pairs was adopted.

### 3.4. Optimization of Magnet Arrangement for Generating a 3D Mixed Magnetic Field

**Figure 8:**
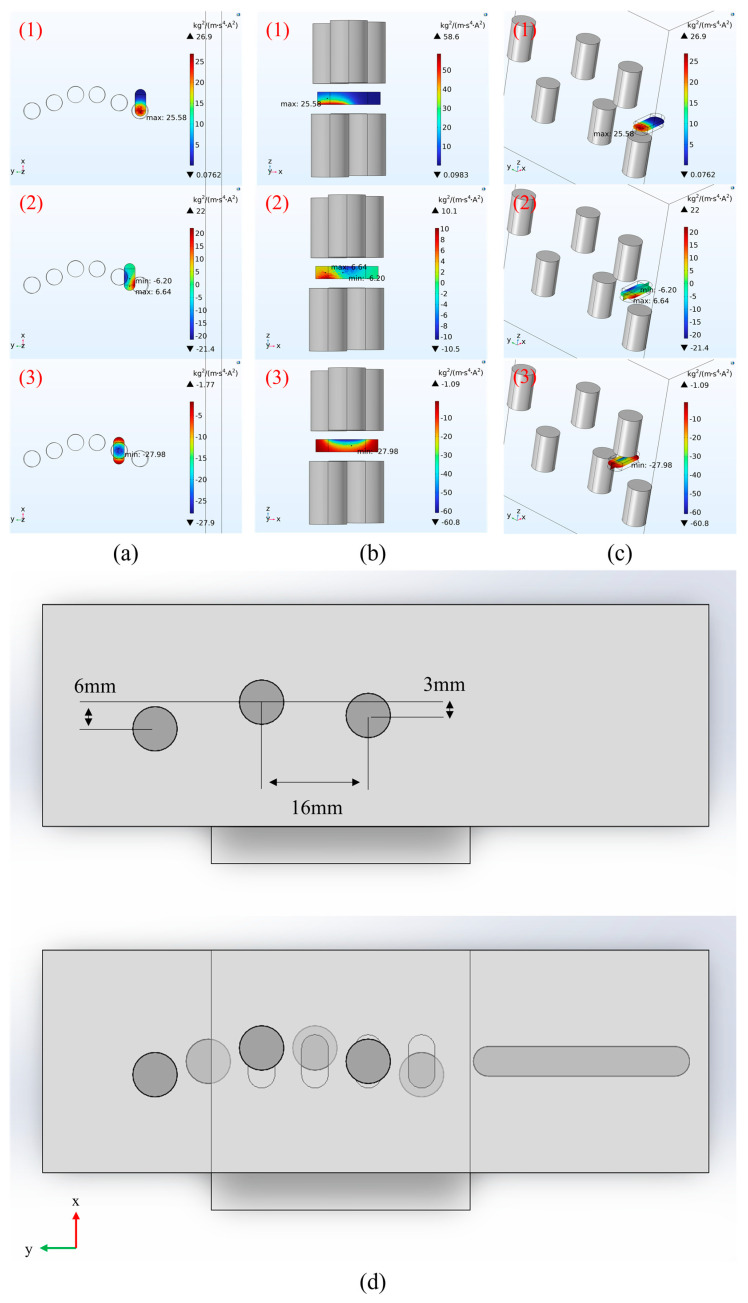
Simulated maximum ***∇B·B*** values along the reaction chamber centerline at different positions within a three-dimensional V-shaped magnetic field array. (**a**) Positions 1−3 in the 3D *xy*-view. (**b**) Positions 1−3 in the 3D *xz*-view. (**c**) Positions 1−3 in the 3D view. (**d**) Optimized arrangement range of the magnets. (For the legends in Figures (**a**)−(**c**), the color gradient from blue to red represents an increase in ***∇B·B*** values. Specifically, more intense blue indicates lower values, while more intense red indicates higher values. The complete set of results for all positions can be found in Figure S4.).

The spatial arrangement of the 3D mixing magnetic field must be precisely matched to the structure of the microfluidic chip’s reaction chamber. Given that the chamber is directly connected to the inlet/outlet channels, the width of the magnetic field requires optimized design: an excessively wide field would overly draw MBs into the channel regions, causing sample loss and uneven mixing; conversely, an overly narrow field would restrict the motion range of the beads. Both scenarios would reduce reaction efficiency. Simulations of the magnet arrangement range were therefore conducted in three-dimensional space to more accurately optimize the magnetic field distribution within the reaction chamber. As indicated previously, under 3D simulation conditions, too small a spacing between magnets along the *x*- and *y*-axes leads to excessive bead aggregation, lowering cell capture efficiency, while too large a spacing creates zones of weak magnetic field where beads stagnate, increasing reaction time. Taking the motion within a single reaction chamber as an example, to achieve a three-dimensional S-shaped trajectory of the beads, the moving magnetic field must vary uniformly across different regions of the chamber. Based on this principle, a periodic field variation pattern was designed as follows: lower left—center—upper right—lower right—center—upper left—lower left. The corresponding magnetic field distribution and the maximum ***∇B·B*** values along the chamber centerline are shown in Figure 8a–c. When a magnet is positioned directly above or below the chamber (positions 1 and 3 in Figure 8a), the generated field is confined to a local area within the chamber. The distribution is uniform and similar to 2D simulation results, with a slight increase in the ***∇B·B*** magnitude. When the field moves such that the chamber lies at the boundary between the fields of two vertically adjacent magnets (position 2 in Figure 8a), the ***∇B·B*** value at the chamber centerline reaches its minimum, as this point corresponds to the interface between the two magnetic fields. Based on the 3D simulation results, using the position directly above or below the chamber center as a reference, other magnet positions were determined as follows: when magnets are positioned at the sides of the chamber, their centers are aligned with the centroids of the chamber’s semi-circular ends; when positioned at the chamber center along its length, the magnet center aligns with the midpoint of the chamber centerline. The magnets were then arranged periodically according to the field distribution pattern. The final magnet arrangement design is shown in Figure 8d. Simulation results for the complete set of positions within one magnetic field cycle are provided in Supplementary Figure S4. 

Based on the results presented in Section 3.3, it can be readily deduced that a shorter distance between the magnet and the chip results in a greater magnetic field gradient value, ***∇B·B***. Consequently, the centerline of the chip’s reaction chamber, being the furthest point from both magnets, corresponds to the position with the minimum ***∇B·B*** value. Provided the **F_m_** at this location exceeds the **F_d_**, the MBs will be attracted and immobilized against one side of the chamber. In actual experiments, the beads are drawn in advance by the magnetic field from the centerline toward the bottom of the chamber, where they are firmly held by the fixed magnet. The **F_m_** acting on the beads at the bottom is significantly greater than the **F_d_** generated during solution exchange; hence, no washout of beads was observed during buffer replacement. These numerical simulation results further corroborate the parameter selection described in Section 2.3, confirming that magnet material, magnet–chamber distance, and spatial arrangement predominantly govern the magnitude and spatial distribution of the magnetic field and its gradient within the reaction chamber.

Although the calculated Re number approaches unity rather than being orders of magnitude smaller than one, in practical operating conditions, magnetic beads may still experience additional hydrodynamic effects, including fluid inertia and wall-induced interactions arising from proximity to the reaction chamber boundaries. These effects can influence bead motion, particularly under conditions of elevated flow rates or reduced bead–wall separation. However, in the numerical simulations conducted in this study, the magnetic beads are immobilized at one side of the reaction chamber under the influence of the external magnetic field, where the magnetic force is significantly larger than the hydrodynamic drag force. Under this force-dominant regime, the contributions of fluid inertia and wall effects become secondary relative to the magnetic confinement. Therefore, to reduce model complexity and computational burden while retaining the dominant physical behavior, these effects were neglected, and the total fluid-induced force acting on the magnetic beads was approximated solely by the viscous drag force described by Stokes’ law [40,41]. Shanko et al. [19,41] also employed a similar simplified model to design a rotating magnet strategy termed magnetic particle swarming (MPS), primarily aimed at non-flow-through systems requiring mixing within initially stagnant fluid. In contrast, the present study focuses on a flow-through system. In practical applications involving the exchange of multiple solutions, the beads not only facilitate fluid mixing but also, through their surface coatings, enable the specific capture of cells, proteins, or nucleic acids for corresponding analytical procedures.

### 3.5. Comparison of CD4^+^ Cell Capture Efficiency Under Different Mixing Strategies

**Figure 9:**
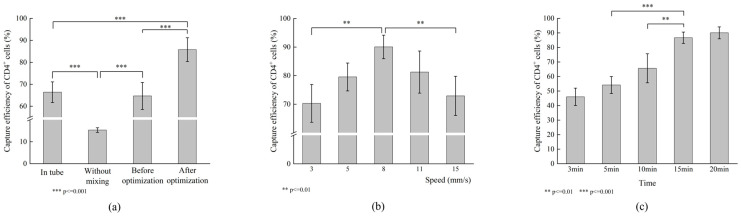
Capture efficiency of CD4^+^ cells by MBs under different conditions. (**a**) Comparison of cell capture rates for different mixing methods. (**b**) Comparison of cell capture rates at different magnetic field actuation speeds. (**c**) Comparison of cell capture rates for different mixing durations. (The corresponding data are provided in Supplementary Figure S5).

Based on the aforementioned simulations, an optimized V-shaped 3D mixing magnetic field was constructed using N52 NdFeB magnets (6 mm in diameter, 10 mm in height) with an inter-layer spacing of 5.4 mm and arranged as described in Section 3.4. This magnetic configuration was then applied to evaluate liquid mixing performance through CD4^+^ cell capture efficiency experiments. Conventional tube-based mixing was used as the control, while the experimental groups included no mixing, pre-optimized magnet arrangement, and post-optimized magnet arrangement. The same cell sample was evenly divided into four portions, with a consistent bead-to-cell ratio of 100:1 (all subsequent experiments used this ratio) and a mixing time of 20 min. As shown in Figure 9a, the cell capture efficiencies for the conventional tube control and the pre-optimized magnetic arrangement were 66.40 ± 4.71% and 64.68 ± 6.15%, respectively, with no significant difference between them (*p* > 0.05, *n* = 12). The no-mixing group showed a markedly lower capture efficiency of only 15.32 ± 1.05%. In contrast, the post-optimized magnetic arrangement increased the capture efficiency to 85.76 ± 5.43%, which differed extremely significantly from both the control and the other experimental groups (*p* < 0.001, *n* = 12). Data were collected from four independent reaction chambers across three chips from different fabrication batches, yielding twelve independent measurements. These results demonstrate that the optimized magnetic field effectively enhances cell capture efficiency in a stable and reproducible manner.

### 3.6. Effect of Magnetic Field Actuation Speed on CD4+ Cell Capture Efficiency

Although simulation results indicated the presence of a relatively weak field region at the transition zone between two alternating magnets, stronger field regions generated by adjacent magnets on either side remained capable of attracting the beads. However, the field strengths produced by the two magnets were numerically close; for example, at position 2 in Figure 8c, the maximum ***∇B·B*** values from the two magnets were 22 and 21.4, respectively. The actual bead motion under such conditions required experimental verification. Furthermore, in practical operation, the magnetic field is continuously translated in one dimension, and its translation speed directly influences bead dynamics. The dwell time of the field at transition zones is also speed-dependent. Therefore, five different magnetic field translation speeds—3, 5, 8, 11, and 15 mm/s—were tested. After 20 min of mixing, the CD4^+^ cell capture efficiencies were compared, as shown in Figure 9b. At a translation speed of 8 mm/s, the capture efficiency reached 90.04 ± 4.11%, representing the highest value. Capture efficiency decreased when the speed was either increased or decreased. The result at 8 mm/s showed a highly significant difference (*p* < 0.01, *n* = 4) compared with those at 3 mm/s (70.27 ± 6.62%) and 15 mm/s (72.91 ± 6.87%). These findings are consistent with experimental observations. Given that the distance between adjacent reaction chambers is 4 mm, at a speed of 3 mm/s the transition time between neighboring magnets exceeded 1 s, and the period of a full cycle was approximately 13.33 s. Under these conditions, beads exhibited noticeable stagnation and aggregation within the chamber, leading to insufficient mixing. When the speed was increased to 15 mm/s, the alternation between magnets became too rapid: the transition time between two magnets was about 0.27 s, and a full cycle took only about 2.67 s. As a result, the beads did not have sufficient time to respond to the magnetic force from an adjacent magnet before being swept into the next region. For instance, at this speed, beads moved directly from the initial lower-left position to the upper-right position and then quickly back to the lower-left position, oscillating only between these two locations without achieving adequate overall motion or mixing, which consequently reduced cell capture. At speeds of 5 mm/s and 11 mm/s, capture efficiencies were 79.54 ± 4.87% and 81.24 ± 7.37%, respectively. Although lower than that at 8 mm/s, the differences were not statistically significant (*p* > 0.05, *n* = 4). Based on these results, a magnetic field translation speed of 8 mm/s was selected for subsequent experiments.

### 3.7. Effect of Mixing Duration on CD4^+^ Cell Capture Performance

To accommodate the workflow of IF staining for flow cytometry and minimize the overall staining time, the effect of mixing duration on cell capture efficiency was investigated. In the initial experimental design, mixing times of 5, 10, 15, and 20 min were selected as evenly spaced intervals to systematically evaluate the influence of mixing duration. Based on the results obtained from these experiments and with the aim of further reducing the mixing time during the washing steps, an additional shorter time point of 3 min was subsequently included. Therefore, mixing times of 3, 5, 10, 15, and 20 min were tested. As presented in Figure 9c, the capture efficiency increased significantly with longer mixing times within the 3 to 15 min range (*p* < 0.01, *n* = 4). However, no statistically significant difference was observed between 15 min (86.66 ± 3.89%) and 20 min (90.04 ± 4.11%) (*p* > 0.05, *n* = 4), indicating that the efficiency had reached a plateau. To further substantiate this plateau behavior, an intermediate mixing time of 17.5 min was evaluated, yielding a capture efficiency of 86.10 ± 2.48%, which showed no statistically significant difference compared with the results at 15 and 20 min. The corresponding data is provided in Supplementary Figure S5. Therefore, in the cell capture step of the automated intracellular IF staining workflow, a mixing duration of 15 min was determined to be optimal, balancing capture efficiency and overall process time.

In this study, Dynabeads™ M-280 magnetic beads with a diameter of 2.8 μm were employed. The beads are coated with streptavidin, enabling specific binding to biotinylated anti-CD4 antibodies; therefore, the capture performance was evaluated exclusively based on CD4^+^ T lymphocytes. Under the optimized conditions, a maximum capture efficiency of 86% was achieved. Although this value does not represent the theoretical upper limit of immunomagnetic separation, it corresponds to an improvement of approximately 20% compared with conventional protocols, which is consistent with our group’s previous findings [36]. Similarly, numerous studies have reported capture efficiencies exceeding 85% for specific cell types by optimizing parameters such as magnetic bead surface functionalization [42], bead concentration and size [43,44], as well as flow rates and microfluidic chip geometries [45,46]. In some cases, leukocyte capture efficiencies as high as 99% have been achieved [47]. However, in most of these approaches, magnetic beads are pre-incubated with cells for 30 min to 1 h prior to on-chip processing, followed by the application of a static external magnetic field to immobilize bead–cell complexes for capture efficiency calculation. Fundamentally, these strategies rely on conventional immunomagnetic separation rather than on-chip mixing enhancement.

In contrast, the present study focuses on dynamic three-dimensional magnetic actuation to induce efficient 3D mixing of magnetic beads within the microfluidic reaction chamber. The primary objective is to enhance bead–cell interactions through improved mixing efficiency, thereby increasing cell capture rates directly on-chip. Our results demonstrate that a mixing duration of only 15 min is sufficient to achieve capture efficiencies comparable to those obtained using conventional pre-incubation protocols requiring 30 min to 1 h [43,44]. Notably, only a limited number of studies have reported microfluidic platforms capable of performing intracellular IF staining at the single-cell level. One of the closest examples is Takahashi et al. [48], in which lymphocytes were physically trapped using a specially designed microstructured array, followed by sequential reagent exchange to complete γH2AX staining and qualitative fluorescence imaging. Compared with that approach, the present system does not rely on complex cell-trapping microstructures and enables automated intracellular IF staining through magnetically enhanced mixing, while additionally allowing quantitative analysis of dose-dependent γH2AX expression using flow cytometry. Together, these results demonstrate that dynamic 3D magnetic mixing provides an effective and scalable strategy for accelerating cell capture and enabling fully automated, cell-level intracellular IF workflows on-chip.

### 3.8. Flow Cytometric Validation of Automated γH2AX Intracellular IF Staining in Captured CD4^+^ Cells

**Figure 10:**
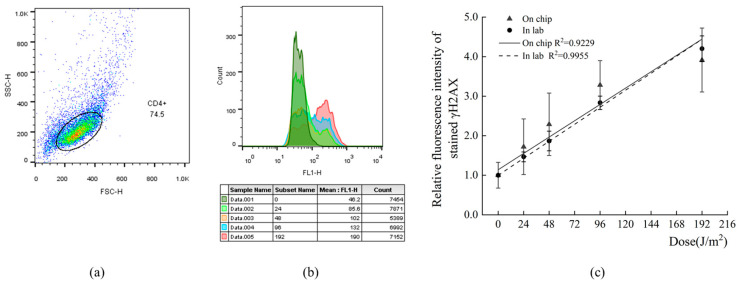
Flow cytometric analysis of γH2AX IF in CD4^+^ cells processed on the microfluidic chip. (**a**) Representative FSC-SSC scatter plot of the CD4^+^ cells in flow cytometric analysis. Each colored dot represents the data for a single cell. The circle indicates the gating strategy, where the population within the gate is selected for further analysis. All samples were analyzed using the same gating strategy as shown here. (**b**) Representative FL1-H fluorescence histograms of γH2AX-stained CD4^+^ cells. (**c**) Dose–response curves of γH2AX fluorescence obtained by conventional in-lab and microfluidic staining on-chip methods.

Based on the optimal 3D mixing conditions determined in the preceding experiments, automated IF staining of the radiation-induced DNA damage marker γH2AX in UVC-irradiated and fixed CD4^+^ cells was successfully achieved using the experimental procedure and setup described in Section 2.5. The fluorescence intensity of the labeled cells was subsequently measured by flow cytometry at the single-cell level. The MFI of the gated CD4^+^ cell population was analyzed to calculate the RFI, and a radiation dose–response relationship was established. Figure 10a illustrates the gating strategy applied to the flow cytometry data, while Figure 10b shows the overlaid fluorescence histograms of FL1-H for a representative experimental sample. Figure 10c compares the fitted dose–response curves obtained using the conventional tube-based staining method and the microfluidic chip-based automated staining approach. The results demonstrate that, within the investigated dose range, samples processed on the microfluidic chip exhibit a clear dose-dependent increase in γH2AX fluorescence, which can be well described by a linear model (solid line, R^2^ > 0.9). This linear response trend is comparable to that obtained using the conventional laboratory staining protocol (dashed line, R^2^ > 0.99), indicating that automated IF staining of intracellular γH2AX in CD4^+^ cells using the microfluidic chip system is feasible and provides reliable quantitative performance. The primary objective of this study is methodological validation rather than exhaustive biological characterization.

The optimized experimental workflow demonstrates a significant reduction in processing time through the strategic allocation of mixing durations. In contrast to the 15 min mixing required for optimal cell capture, a brief 3 min interval was found to be sufficient for the washing and reagent-exchange steps. This reduction is attributable to the high mixing efficiency of the 3D magnetic field, which ensures rapid interfacial contact between the captured cells and the solution. To achieve high sensitivity and robust signal separation in flow cytometry, the antibody incubation time was maintained at 30 min to ensure binding saturation. A key innovation of our platform lies in the use of a dynamically actuated 3D magnetic field, which serves a dual purpose: acting as a “virtual centrifuge” for cell retention and an active mixer for enhanced kinetic reactions. This integration allows for the simplification of the washing sequence and the unification of permeabilization and staining into a single, consolidated step. Consequently, the proposed method completes the entire IF staining process within 1.5 h, achieving a significant time reduction compared to the 3–6 h required for conventional manual procedures [2]. This automated system offers a high degree of efficiency and autonomy, substantially reducing the workload of reagent exchange and centrifugal enrichment. Such features are particularly advantageous for deployment in resource-constrained or remote settings, including subsea laboratories and orbital space stations, where traditional laboratory infrastructure is unavailable.

In this context, it is important to note that the present work differs fundamentally from most reported IMB-based bioanalytical assays, which primarily employ magnetic fields as static tools for target immobilization and enrichment. Here, the dynamically actuated 3D magnetic field is deliberately integrated as an active on-chip processing element to enable accelerated mixing, washing, and intracellular immunofluorescence staining at the single-cell level. The majority of bioanalytical assays based on MBs reported to date rely on static magnetic fields to immobilize beads for target separation and purification. For example, Jessica et al. [49] isolated and enriched circulating tumor cells (CTCs) from whole blood using immunomagnetic separation, followed by IF labeling for imaging and analysis, where the primary function of MBs remained separation rather than on-chip processing. Gao et al. [50] developed a method combining immunomagnetic enrichment with lateral flow immunochromatography (LFIC) for the identification of genogroup II human norovirus (HuNoV) in fecal samples. In addition, Martina et al. [51] implemented an IMB–based assay integrating LFIC and CL aboard the International Space Station to quantify cortisol levels in astronauts’ saliva, achieving results consistent with ground-based measurements. In contrast, the experimental results of the present study demonstrate the feasibility of performing automated intracellular IF staining using a dynamically actuated magnetic field–microfluidic chip cooperative architecture. Compared to our previous implementations, the present study further advances the platform by replacing the earlier magnet configurations with a simplified one-dimensional actuation strategy and by redesigning the microfluidic architecture to enable more robust on-chip processing and automated intracellular immunofluorescence workflows. Importantly, although γH2AX was selected as a representative validation target in this work, the proposed design is not limited to a specific biomarker. By employing MBs functionalized with different ligands, the platform can be readily adapted for capturing various cell types and analyzing diverse intracellular biomarkers.

Moreover, while numerous studies have focused on the automated and integrated preprocessing of complex biological samples, such as blood, the emphasis of this work is placed specifically on the automation of intracellular IF staining at the cellular level. The proposed platform is designed to be compatible with upstream sample preprocessing modules via standard fluidic interfaces and could be combined, in future implementations, with on-chip flow cytometric detection approaches. Such modular integration highlights the potential of the proposed system as a key enabling component for POCT-oriented analytical workflows targeting biomarkers that require complex intracellular IF staining procedures, including radiation-induced biomarkers such as γH2AX. In future work, automated IF staining workflows for multiple cell types will be systematically evaluated, and additional on-chip detection functionalities will be explored to further advance toward a more unified microfluidic analytical system.

## 4. Conclusions

This study developed an automated on-chip intracellular IF staining system enabled by a 3D magnetic mixing scheme. By coupling 1D magnet actuation with a specific microfluidic architecture, we achieved highly efficient 3D periodic bead motion for enhanced mass transfer and cell retention. Numerical simulations provided critical design parameters for magnetic gradients and fluidic shear balances, enabling the optimization of magnet arrangement and flow kinetics. Experimental validation demonstrated a significant enhancement in performance, with a CD4^+^ cell capture efficiency of 86%—a nearly 20% improvement over conventional manual protocols. Furthermore, the platform successfully automated the intracellular staining workflow of the nuclear protein γH2AX within 1.5 h, yielding results consistent with standard laboratory assays and maintaining a robust dose–response linearity. The automated single-chamber protocol not only reduces processing time but also minimizes manual intervention. As a key enabling module for automated intracellular staining, the proposed system shows strong potential to be incorporated into future biosensing and POCT-oriented analytical workflows targeting biomarker detection in resource-limited or constrained environments. It should be noted that, in the present study, each chip was used for a single sample with multiple replicates. In future work, we plan to improve the chip design to enable simultaneous analysis of multiple samples, as well as to extend the system to different cell types and various biomarkers.

## Figures and Tables

**Table 1:** The series of parameters designed in this article.

Parameters		Value	Unit
Fluid velocity	v	50, 100, 150, 200	μL/min
Magnetic field	Magnet material	N35, N45, N52	-
	Magnet height (H)	4, 6, 8, 10, 12	mm
	Magnet distance (D)	0.5, 1.0, 1.5, 2.0, 2.5	mm

**Table 2:** The main parameters and settings of the physics interfaces.

Physics Interfaces	Major Parameters		Value	Unit
SPF	Fluid properties	Default discretization	P1 + P1	-
		Density	1000	Kg/m^3^
		Dynamic viscosity	0.001	Pa·s
	Boundary condition	wall	No slip	-
		Inner inlet flow rate	0.83 × 10^−9^–3.33 × 10^−9^	m^3^/s
		Outlet pressure	0	Pa
MFNC	Relative permittivity		1	1
	Magnet material		N35, N45, N52	NdFeB (material)
	Magnet height (H)		4–12	mm
	Magnet distance (D)		0.5–2.5	mm
	Magnetic flux density		B = μ_0_μ_r_H + B_r_	T

**Table 3:** Maximum values of **Re**, **F_d_**, and ***∇B·B*** at different flow rates.

Flow Rates μL/min	Re	F_d_ pN	*∇B·B* kg^2^/(m·s^4^·A^2^)
50	1.01	33.45	2.76
100	2.05	67.84	5.61
150	3.14	103.82	8.58
200	4.30	142.10	11.75

## Data Availability

The data that support the findings of this study are available from the corresponding author upon reasonable request.

## Supplementary Materials

The following supporting information can be downloaded at: https://www.mdpi.com/article/10.3390/bios16020120/s1, Figure S1: Simulation results of ***∇**B·B*** of N35 magnet on the centerline of the reaction chamber at different Distances (D) and Heights (H); Figure S2: Simulation results of ***∇**B·B*** of N45 magnet on the centerline of the reaction chamber at different Distances (D) and Heights (H); Figure S3: Simulation results of ***∇**B·B*** of N52 magnet on the centerline of the reaction chamber at different Distances (D) and Heights (H); Figure S4: Simulation of the maximum ***∇**B·B*** along the centerline of the chip reaction chamber at different positions in a 3D V-shaped magnetic field. Figure S5: Comparison of cell capture rates for different mixing durations.
