# Supplementary material for: Automated Intracellular Immunofluorescence Staining Enabled by Magnetic 3D Mixing in a Modular Microfluidic Platform
Source: Biosensors (Basel). 2026 Feb 13;16(2):120. doi: 10.3390/bios16020120 (PMC12938186; doi:10.3390/bios16020120)
Supplement: Supplementary file 1 [file biosensors-16-00120-s001.zip › biosensors-4112676-supplementary.pdf]

Article

Automated Intracellular Immunofluorescence Staining Enabled by Magnetic 3D Mixing in a Modular Microfluidic Platform

Zhengyi Zhang [1], Mengyu Wang [1], Runtao Zhong [1], Yingbo Zhao [1,†] and Yeqing Sun [1,*]

[1] Institute of Environmental Systems Biology, College of Environmental Science and Engineering, Dalian Maritime University, Dalian 116026, China; zhengyi-zhang@dlmu.edu.cn (Z.Z.); wangmengyu@dlmu.edu.cn (M.W.); rtzhong@dlmu.edu.cn (R.Z.)

† Illumina China Scientific Co., Ltd., Beijing 100000, China; yingbo.zhao@illumina.com

* Correspondence: yqsun@dlmu.edu.cn

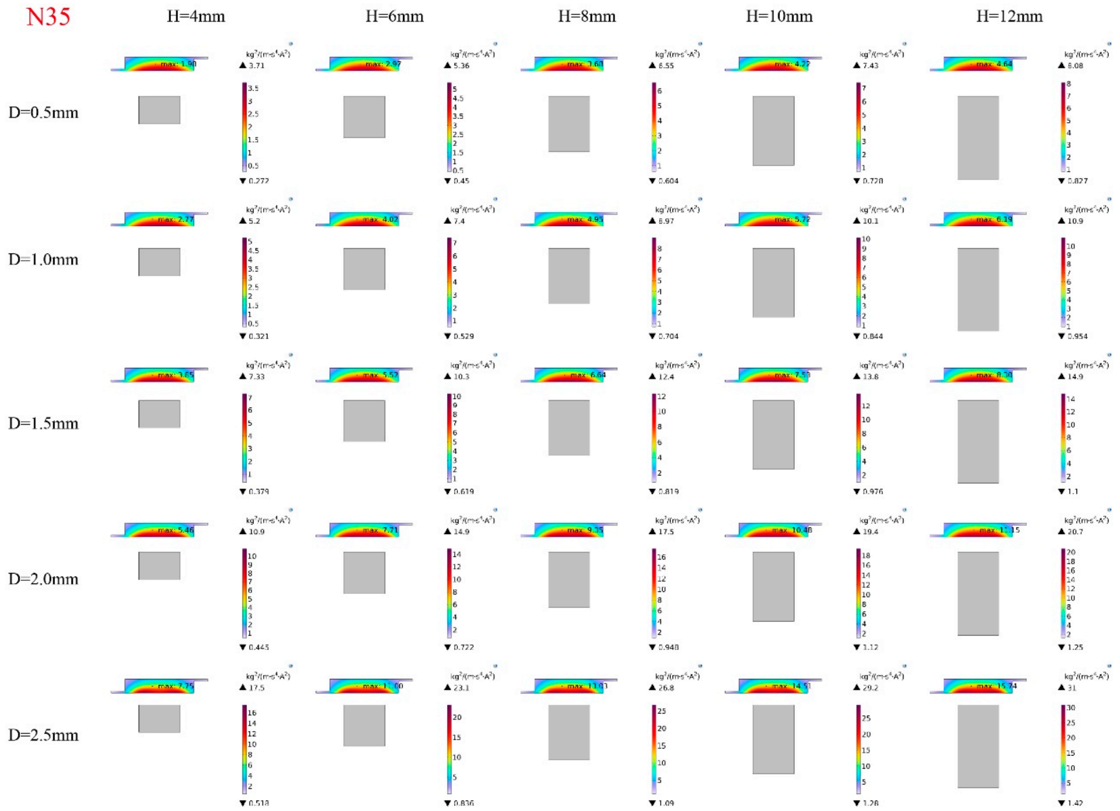

Figure S1. Simulation results of $\nabla B \cdot B$ of N35 magnet on the centerline of the reaction chamber at different Distances(D) and Heights(H)

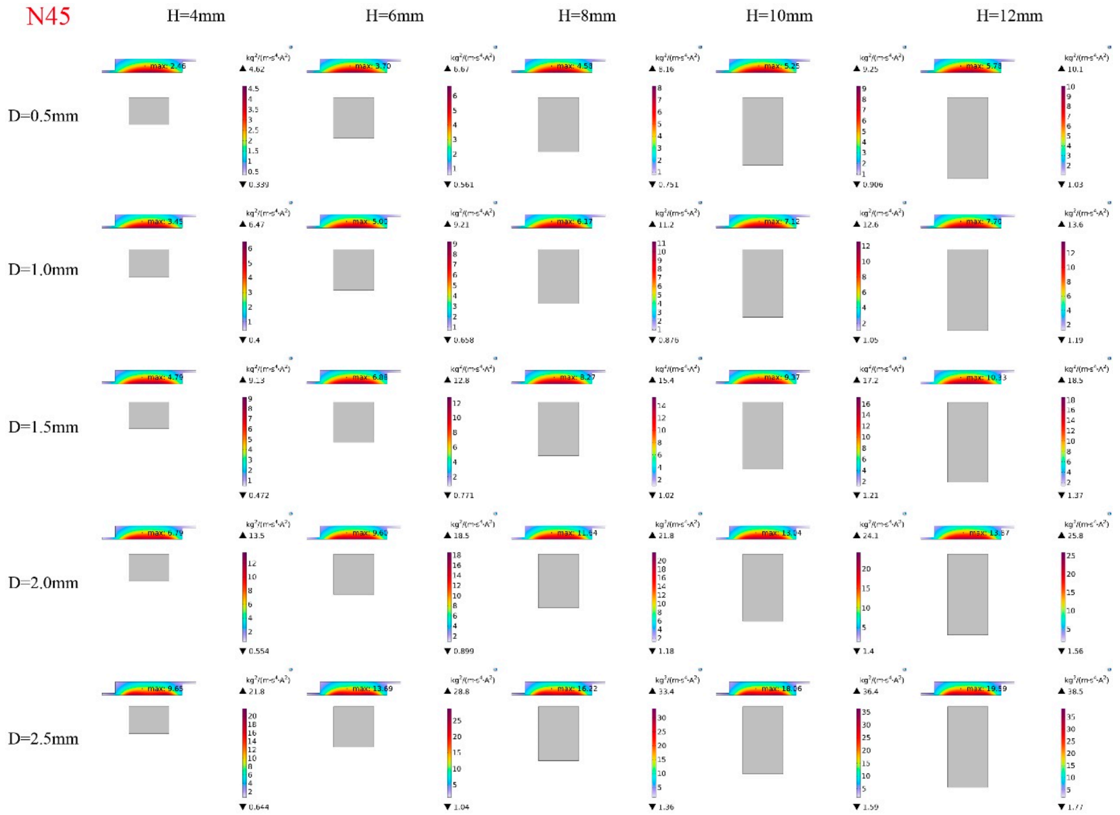

Figure S2. Simulation results of $\nabla B \cdot B$ of N45 magnet on the centerline of the reaction chamber at different Distances(D) and Heights(H)

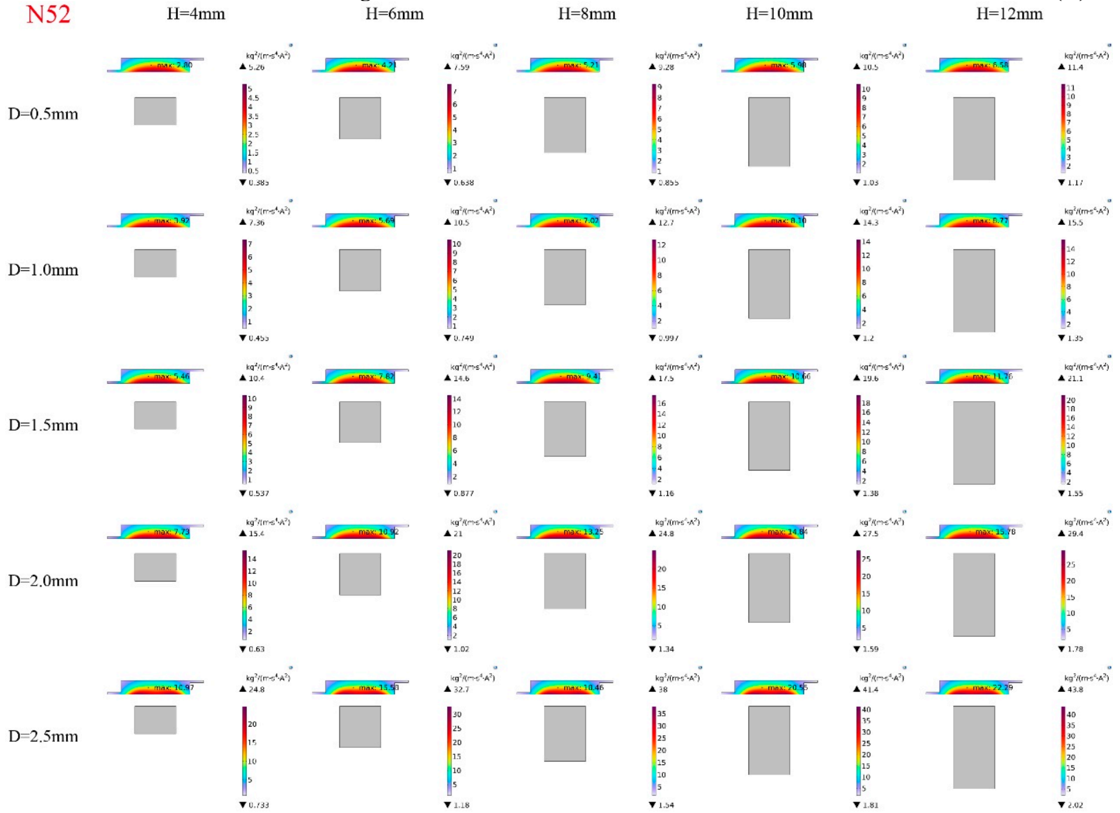

Figure S3. Simulation results of $\nabla B \cdot B$ of N52 magnet on the centerline of the reaction chamber at different Distances(D) and Heights(H)

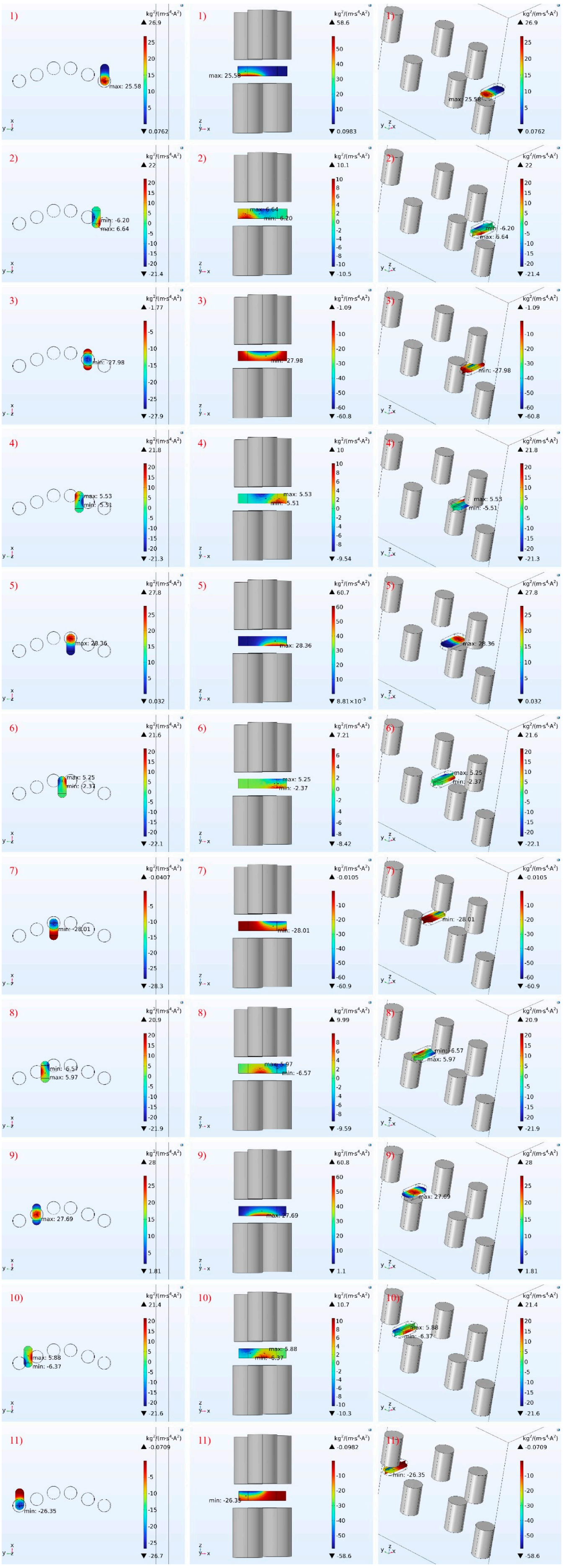

Figure S4. Simulation of the maximum $\nabla B \cdot B$ along the centerline of the chip reaction chamber at different positions in a 3D V-shaped magnetic field.

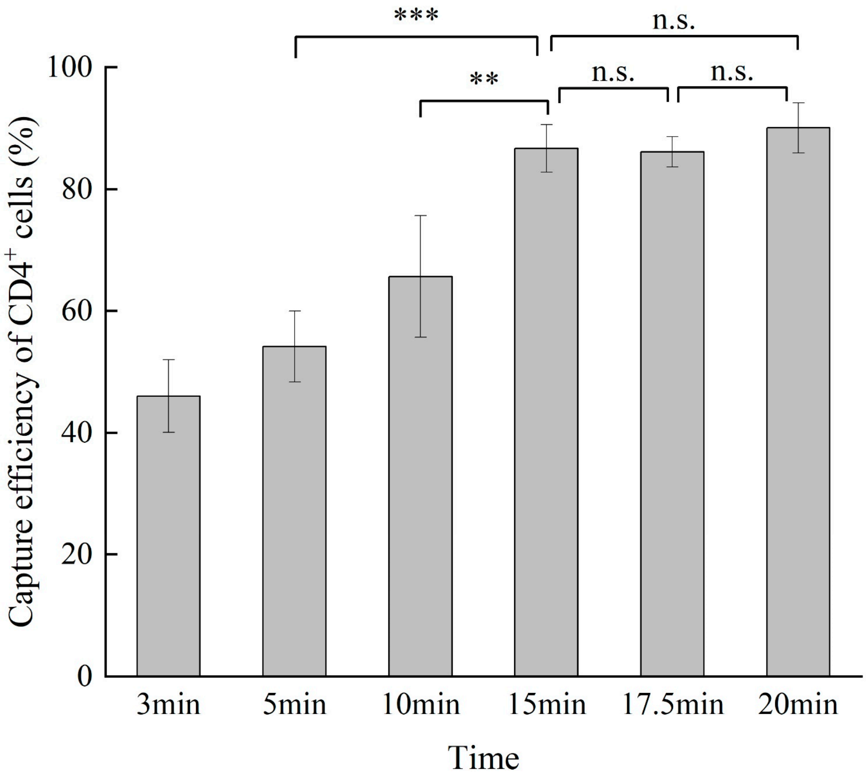

* p<=0.05 ** p<=0.01 *** p<=0.001

Figure S5. Comparison of cell capture rates for different mixing durations. 3min (46.02 ± 5.97%), 5min (54.13 ± 5.83%), 10min (65.65 ± 9.97%), 15 min (86.66 ± 3.89%), 17.5 min (86.10 ± 2.48%) and 20 min (90.04 ± 4.11%).
